# The assessment of breast cancer biomarkers in diagnosis, prognosis and treatment monitoring: integrated analysis

**DOI:** 10.1007/s00432-025-06271-1

**Published:** 2025-08-22

**Authors:** Patrycja Królewska-Daszczyńska, Aleksandra Englisz, Maria-Laura Morawiec, Joanna Miśkiewicz, Maciej Gołębski, Aleksandra Mielczarek-Palacz

**Affiliations:** Department of Immunology and Serology, Faculty of Pharmaceutical Sciences in Sosnowiec, Medical University of Silesia, 40-055 Katowice, Poland

**Keywords:** Breast cancer, Biomarkers, Molecular markers, Biomarkers combination, Personalised treatment

## Abstract

Breast cancer is a heterogeneous disease, which is still a challenge for modern cancer diagnostics. Despite significant progress in diagnosis, monitoring and treatment of breast cancer, it remains the leading cause of cancer-related death in women. Effective screening methods, which enable early diagnosis of the disease and rapid personalised treatment are crucial to improving survival of women with breast cancer. In recent years, increasing attention has been paid to the clinical utility of circulating biomarkers, such as proteins, autoantibodies, miRNAs, circRNAs, ctDNA or CTCs, which have the potential to supplement traditional methods of BC diagnosis. Despite much research, no sufficiently sensitive and minimally invasive marker has been identified to aid in the early diagnosis, monitoring of disease progression and response to therapy in women with breast cancer. Combinatorial analysis of circulating biomarkers is novel and promising approach, which may overcome the limitations of single biomarker assays.

## Introduction

Female breast cancer (BC) was the second most commonly diagnosed cancer (2.3 million new cases; 11.6% of all cancer patients) and the fourth leading cause of cancer mortality ( 665 684 deaths) worldwide in 2022 (Bray et al. 2024). Most of BC cases occur in transitioned countries, however mortality rates are higher in transitioning versus transitioned countries (Bray et al. 2024; Arnold et al. 2022). In European countries, BC was estimated to be the most frequently diagnosed cancer in female population and the first cause of cancer mortality in women for 2020 (Dyba et al. 2020). Moreover, differences in the five-year survival rates for BC are very wide among countries. For women diagnosed during 2010–2014, the five-year net survival approached 90% in United States, while in India it was 66% (Allemani et al. 2018).

The global differences in BC incidence are associated with human development and various risk factors for the disease (Wilkinson and Gathani 2022). Younger age of menarche or late menopause age, having fewer children and not breastfeeding or breastfeeding only for a short period of time are among risk factors for BC development (Wilkinson and Gathani 2022; Collaborative Group on Hormonal Factors in Breast Cancer 2012, 2002). Moreover, other BC risk factors include obesity, alcohol consumption, increasing life expectancy and changes in female reproductive patterns (Wilkinson and Gathani 2022). For less developed countries, however, the reasons that may correlate with the lower BC incidence and higher mortality include low BC awareness and limited healthcare resources reflecting lack of screening programs, late diagnosis at an advanced stage and decreased access to proper treatment (Wilkinson and Gathani 2022; Tfayli et al. 2010; Costa Vieira et al. 2017).

Breast cancer is a histopathologically and genetically heterogeneous disease which is reflected by histopathologic and molecular classifications and staging system. Breast cancers differ among different patients as well as within an individual tumour, due to the presence of heterogeneous cell populations (Turashvili and Brogi 2017). Moreover, breast tumours are diverse in terms of growth intensity, aggressiveness, metastatic imprint and response to therapy (Barba et al. 2021; Beňačka et al. 2022).

Early diagnosis of BC and adequate screening are crucial for improving the survival of patients and effective treatment. Currently, the diagnosis of BC is mostly based on imaging techniques, among which mammography is the primary screening method, and, histopathological confirmation on tissue biopsy samples (Barba et al. 2021; Wang 2017; Jafari et al. 2018; Pesapane et al. 2020). However, despite evidence that screening mammography reduces BC mortality (Broeders et al. 2012; Nickson et al. 2012) this method has also some limitations (Gegios et al. 2023), such as reduced sensitivity of the mammography examination in women with dense breast tissue (McLean and Stone 2018; Checka et al. 2012; Destounis et al. 2017; Gils et al. 1998). Moreover, tissue biopsy, which is required after the detection of a suspected lesion in the breast, is an invasive and time-consuming procedure that may not reveal the complete heterogeneity of cancer (Eigeliene et al. 2019; Zubor et al. 2019).

In recent years, multiple molecular test have been developed to supplement traditional clinicopathological prognostic factors for BC, such as lymph node metastasis, tumour size and tumour grade (Nicolini et al. 2018). Currently, the aim of many studies is to search for novel minimally invasive biomarkers useful for the diagnosis of early-stage BC, prognosis, monitoring response to treatment and prediction of tumour progression (Pesapane et al. 2020; Ozawa et al. 2020).

This review critically evaluates recent advances in BC biomarkers, with special emphasis on circulating biomarkers. Particular attention was paid to the combinatorial analysis of biomarkers, which may be a modern approach to overcome the limitations of single biomarker assays.

## Classical markers in breast cancer diagnosis

### Molecular markers defining molecular subtypes of breast cancer

The molecular classification of breast cancer reflects differences in the clinicopathological features and response to treatment of tumour subtypes (Roy et al. 2023). Classical molecular biomarkers, detected from tumour cells by immunohistochemistry, that define molecular subtypes include oestrogen receptor (ER), progesterone receptor (PR) and human epidermal growth factor receptor 2 (HER2) (Barba et al. 2021; Beňačka et al. 2022; Dai et al. 2016). The expression of these biomarkers should be assessed routinely in all newly diagnosed breast carcinomas as they are established prognostic and predictive factors critical in guiding appropriate patient treatment (Turashvili and Brogi 2017; Wolff et al. 2023; Allison et al. 2020; Grogan Fleege and Cobain 2021; Jorns 2019).

#### Estrogen and progesterone receptors

ER and PR are nuclear receptors belong to the steroid/nuclear receptor family of ligand-activated transcription factors (Jameera Begam et al. 2017; Hilton et al. 2018). There are two isoforms of ER encoded by independent genes located on different chromosomes: ERα (*ESR1* at 6q24-27) and ERβ (*ESR2* at 14q22-24) (Xia and Lin 2022; Khan et al. 2022; Clusan et al. 2021). In BC cells ERα shows higher expression than ERβ and, being involved in the regulation of endocrine function, is responsible for growth, survival and proliferation of breast epithelial cells, leading to tumour development (Almeida 2020). PR has two isoforms: PR-A and PR-B encoded by *PGR* gene located on chromosome 11 (Krystel-Whittemore et al. 2024; Boonyaratanakornkit et al. 2018; Tarighati et al. 2023; Wu et al. 2020). The expression of PR is regulated by ERα and the presence of PR indicates that ER pathway is functional (Allred 2010; Rakha et al. 2022) indicating the potential sensitivity to endocrine therapy (Tarighati et al. 2023; Neves Rebello Alves et al. 2023).

ER is a well-established predictive marker in BC used for consideration of endocrine therapy (Rakha et al. 2022) that for ER-positive patients include aromatase inhibitors (AI), which inhibit the estrogens synthesis and anti-estrogens: selective ER modulators (SERMs; e.g., tamoxifen) and selective ER downregulators (SERDs; e.g., fulvestrant), which interfere with estrogen-dependent pathways (Nicolini et al. 2018; Almeida 2020; Awan and Esfahani 2018; Hanker et al. 2020).

It was demonstrated that patients with ER-positive/PR-positive BCs were more likely to benefit from endocrine therapy than those with ER-positive/PR-negative tumours (Nordenskjöld et al. 2016; Bardou et al. 2003). Meta-analysis by Zhong et al. (2022) showed that patients with high expression of these hormone receptors have better prognosis. However, meta-analysis of randomized trials revealed that PR measurements did not seem to be predictive of endocrine therapy efficacy (Early Breast Cancer Trialists’ Collaborative Group 2011). Thus, the predictive role of PR independent of ER is debatable.

However, the Expert Panel recommends routine PR testing in invasive BCs and points to the prognostic role of PR (Allison et al. 2020). ER-positive/PR-negative tumours were found to have more aggressive features than ER-positive/PR-positive BCs (Arpino et al. 2005) Moreover, meta-analysis by Shiino et al. (2022) revealed that in recurrent tumours the loss of ER or PR is associated with shorter overall survival compared with receptor-positive concordant groups.

Distant metastases and resistance to therapy are the main causes of mortality in BC patients (Szostakowska et al. 2019). ER+ BCs may develop recurrences in distant organs, which can be detected years after the primary tumour diagnosis and adjuvant therapy, what suggest the dormancy of ER+ cancer cells (Zhang et al. 2013; Clarke et al. 2015). Moreover, it was shown that during the five years after primary BC diagnosis patients with ER-negative tumours have a higher risk of second BC (Lowry et al. 2023), whereas, for patients with ER-positive tumours the risk of recurrence is higher beyond five years in comparison with ER-negative cancers (Colleoni et al. 2016).

#### Human* epidermal growth factor receptor 2*

HER2 (HER2/*neu*, ErbB2) is a member of human epidermal growth factor receptor family. The HER2 protooncogene (*neu*, c-erbB-2) is located on chromosome 17q21 and encodes the transmembrane HER2 protein (Yarden 2001; Moasser 2007). HER2 testing should be performed in all newly diagnosed and metastatic BC patients. The techniques used to determine HER2 status (positive, equivocal, negative) include immunohistochemistry (IHC) for protein overexpression and/or in situ hybridization (ISH) for gene amplification (Wolff et al. 2023; Wolff et al. 2018; Rakha et al. 2022).

HER2 gene is amplified or the protein is overexpressed in approximately 15% of BC patients (Rakha et al. 2022; Taucher et al. 2004; Wf et al. 2014). HER2 overexpression promotes tumour growth by activating MAPK and PI3K/AKT signalling pathways leading to enhanced cell proliferation, invasion and metastasis (Beňačka et al. 2022; Nicolini et al. 2018; Pan et al. 2024). In BCs patients, HER2 amplification/overexpression is associated with poor prognosis (Ménard et al. 2001). HER2/neu positive expression was reported to be associated with decreased survival in BC patients (Cao et al. 2007).

HER2-targeted therapy significantly improved the outcome for HER2-positive BC patients (Harbeck 2022; Martínez-Sáez and Prat 2021). HER2-targeted therapies include monoclonal antibodies (trastuzumab, pertuzumab), tyrosine kinase inhibitors (lapatinib, tucatinib, neratinib, pyrotinib) and antibody–drug conjugates (ADC) (Stanowicka-Grada and Senkus 2023; Dempsey et al. 2023).

### Additional biomarkers

Ki67 (encoded by *MKI67*) is a nuclear antigen that is a marker associated with cell proliferation (Zhang et al. 2021a; Schlüter et al. 1993) and is usually detected by IHC and reported as Ki67 index (Penault-Llorca and Radosevic-Robin 2017). It is mainly used in ER+/HER2- BC to differentiate luminal A from luminal B tumours (Penault-Llorca and Radosevic-Robin 2017). Despite evidence of the prognostic and predictive importance of Ki67 (Zhang et al. 2021a; Petrelli et al. 2015; Nielsen et al. 2021), the analytical validity of this marker is still questionable due to the lack of a standardised procedure of Ki67 assessment and the actual clinical utility (Rakha et al. 2022; Penault-Llorca and Radosevic-Robin 2017).

Cytokeratins belong to intermediate filament proteins of epithelial cells (McGinn et al. 2022; Moar et al. 2023). In BC, the most commonly used assays include CYFRA 21.1—for CK19, tissue polypeptide antigen (TPA)—for CK8, CK18, CK19 and tissue polypeptide-specific antigen (TPS)—for CK8 and CK18 (Moar et al. 2023; Mirabelli and Incornoto 2013; Nicolini et al. 2015).

p53 is a transcription factor and tumour suppressor, encoded by *TP53* gene, involved in the regulation of several cell functions such as cell cycle regulation, apoptosis, senescence and DNA repair (Neves Rebello Alves et al. 2023; Marvalim et al. 2023). In BC, the *TP53* gene is the most frequently mutated gene, being mutated in nearly 30% of all BC cases (Shahbandi et al. 2020). Mutated p53 may serve as a potential biomarker and therapeutic target for BC patients, particularly in triple negative subtype as about 80% of those patients have mutation in p53 (Duffy et al. 2018).

BRCA1 and BRCA2 are two tumour suppressor genes that play a role in DNA repair processes (Neves Rebello Alves et al. 2023; Lee et al. 2020). The pathogenic variants of these genes are associated with hereditary BC that accounts for 5–10% of all BC cases (Lee et al. 2020). Patients with BRCA1 mutation carrier status mainly have triple negative BCs, whereas tumours arising in BRCA2 carriers are more likely to be ER-positive (Talhouet et al. 2020; Mavaddat, et al. 2012). There are evidence of the prognostic role of BRCA1/BRCA2 in BC and the therapeutic target for this disease (Jin et al. 2022). However, there are conflicting data on the predictive and prognostic significance of BRCA mutations on the survival of patients with non-metastatic BC (Neves Rebello Alves et al. 2023; Talhouet et al. 2020).

### Serum biomarkers in clinical use

Serum tumour markers in BC include cancer antigen 15–3 (CA 15–3), carcinoembryonic antigen (CEA), cancer antigen 27–29 (CA27-29) and cancer antigen 125 (CA 125) (Tarighati et al. 2023; Moar et al. 2023; Seale and Tkaczuk 2022). In the clinical fields, the most widely used serum markers in BC are CA 15–3 and CEA (Shao et al. 2015). It was shown that BC patients with elevated preoperative CA 15–3 and CEA levels had worse prognosis (Shao et al. 2015; Uehara et al. 2008; S. Lee et al. 2013). Moreover, serial measurement of CEA and CA15-3 was shown to be useful for early detection of BC recurrence (Molina et al. 1995). CA15-3, CEA and CA27.29 was found to have increased sensitivity in metastatic BC (Hou et al. 1999) and to be useful in monitoring response to therapy with serial measurements (Seale and Tkaczuk 2022). Fang et al. (2017a) demonstrated that high preoperative CA125 levels may predict poor outcome and prognosis in BC patients.

These circulating biomarkers are useful in the management of metastatic BC. In early BC they have prognostic significance but, due to low sensitivity in early disease and lack of specificity, most expert panels do not recommend these markers for screening (Seale and Tkaczuk 2022; Duque et al. 2022).

## Potential markers in breast cancer diagnosis, prognosis and treatment monitoring

### Proteins

#### Galectins

Galectins are a family of lectins, that have an affinity for β-galactoside structures. These proteins are involved in multiple processes such as cell cycle, apoptosis, cell differentiation or RNA transcription as well as cancerogenesis (Delacour et al. 2009).

In BC tissues increased galectin-1 expression was observed in stromal cells of invasive carcinoma compared to carcinoma in situ and the association between expression of galectin-1 in cancer-associated stromal cells and different clinicopathologic parameters, including tumour invasiveness, was demonstrated (Jung et al. 2007). Overexpression of galectin-1 was found in claudin-low BC (Balestrieri et al. 2021). The potential of galectin-1 as a therapeutic target to overcome immunosuppression associated with BC was also studied (Dalotto-Moreno et al. 2013; Chung et al. 2024). Chetry et al. (2022) demonstrated that high mRNA expression of galectin-1 (LGALS1) was associated with a poor prognosis in BC patients, while overexpression of galectin—2 (LGALS2) was related to a better prognosis. Decreased expression of galectin-3 was found to be associated with aggressiveness and poor prognosis in BC (Castronovo et al. 1996; Ilmer et al. 2016; Shafiq et al. 2020). Ilmer et al. (2016) showed that decreased galectin-3 expression was associated with poor survival and advanced locoregional invasion in BC patients. Shafiq et al. (2020) found that elevated galectin-3 levels in stroma are associated with response to chemotherapy in BC. Elevated expression of galectin-7 was found to be restricted to high-grade BCs (Demers et al. 2010). Moreover, overexpression of galectin-7 was shown to increase BC cells ability to metastasise to lungs and bones in mouse models (Demers et al. 2010). Trebo et al. (2020) found that in primary BC patients high expression of galectin-7 in the cytoplasm was correlated with a worse outcome, whereas high expression of galectin-8 was associated with an improved patients outcome. The antimetastatic potential of galectin-9 in BC was revealed by Irie et al. (2005), as the authors found that patients with galectin-9—positive tumours had a lower frequency of distant metastasis and more favourable disease-free survival in comparison with galectin-9—negative tumours, suggesting prognostic potential of galectin-9.

Several studies evaluated serum concentrations of galectins in BC patients. Barrow et al. (2011) showed significantly increased serum concentrations of galectin-2, -3, -4 and -8 and significantly lower concentration of galectin-1 in patients with BC. However, Gurel Cayir et al. (Gurel Cayir et al. 2020) found higher serum levels of galectin-1 in preoperative BC patients compared to the group of healthy controls. Moreover, higher serum galectin-1 level in preoperative patients compared to postoperative patients was shown, what may suggest that serum levels of galectin-1 positively corelate with tumour presence. According to these findings, galectin-1 may have importance in breast carcinogenesis as well as in evaluation response to treatment (Gurel Cayir et al. 2020). Increased serum levels of galectin-1 were also shown in BC patients who received systemic therapy (hormone therapy, immunotherapy or chemotherapy) compared to patients, who were not exposed to treatment (Funkhouser et al. 2023). Blair et al. (2021) found increased circulating concentrations of galectin-1, -3 and -7 in BC patients. However, statistically significant increase in concentration of galectin-3 was observed in all stages, whereas galectin-1 was elevated in stages I and III and galectin-7 only in stage I (Blair et al. 2021). Shafiq et al. (2020) showed that plasma levels of galectin-3 had an inverse correlation with increasing grade of BC. Moreover, the authors found a relationship between elevated levels of galectin-3 and response to chemotherapy. Higher serum levels of galectin-3 in BC patients were also found by Topcu et al. (2018). Additionally, the study showed that between patients with and without metastasis there was no difference in serum levels of galectin-3 (Topcu et al. 2018). In a study by Funkhouser et al. (2023), serum concentrations of galectin-9 was found to be increased in HER2-amplified tissues and decreased in samples with positive hormone receptor markers. In another study, Funkhouser et al. (Funkhouser et al. 2022) showed higher levels of galectins-1, -3, -8, and -9 in serum of BC patients with a mutation in the *KIT* gene. Markalunas et al. (2024) found correlations between *KIT* mutations and elevated serum levels of galectin-9 in BC patients, *FLT3* mutations and lower serum levels of galectin-1 and -9 as well as *TP53* mutations and higher serum levels of galectin-3 in luminal A subtype. Moreover, higher serum galectin-3 levels were shown in patients with invasive ductal carcinoma in comparison with patients with ductal carcinoma in situ. Additionally, elevated serum galectin-3 levels were found in patients with both *TP53* and *PIK3CA* mutations, while there was no difference in galectin-3 levels in patients with one or neither mutation (Markalunas et al. 2024). Summarised information of selected galectins in BC presents Table 1.

**Table 1:** Significance of galectins in tissue and serum in BC

Galectin	Expression in tissue/cell line	Serum/plasma concentration	Proposed clinical utility
Galectin-1	↑ in stromal cells of invasive BC and correlate with tumour invasiveness (Jung et al. 2007) ↑ in claudin-low BC (Balestrieri et al. 2021) ↑association with a poor prognosis (Chetry et al. 2022)	↓ (Barrow et al. 2011) ↑ in preoperative patients (Gurel Cayir et al. 2020) ↑ in patients exposed to treatment (Funkhouser et al. 2023) ↑ (in stage I and III) (Blair et al. 2021) ↑ in patients with *KIT* mutation (Funkhouser et al. 2022) ↓ in patients with *FLT3* mutation in luminal A subtype (Markalunas et al. 2024)	Therapeutic target (Dalotto-Moreno et al. 2013; Chung et al. 2024) Treatment evaluation (Gurel Cayir et al. 2020)
Galectin-2	↑ associated with a better prognosis (Chetry et al. 2022)	↑ (Barrow et al. 2011)	Potential immunotherapy target in TNBC (Ji et al. 2022) Immunotherapy response, drug resistance (He et al. 2023) Immunotherapeutic biomarker, potential therapeutic target (Li et al. 2024)
Galectin-3	↓ associated with aggressiveness and poor prognosis (Castronovo et al. 1996; Ilmer et al. 2016; Shafiq et al. 2020) ↓ associated with advanced locoregional invasion and poor survival (Ilmer et al. 2016) ↑ levels in stroma in relationship with response to chemotherapy (Shafiq et al. 2020)	↑ (Barrow, et al. 2011; Blair et al. 2021; Topcu et al. Sep. 2018) inverse relationship with increasing grade (Shafiq et al. Mar. 2020) ↑ in relationship with chemotherapy response (Shafiq et al. Mar. 2020) ↑ in patients with *KIT* mutation (Funkhouser et al. 2022) ↑ in patients with both *TP53* and *PIK3CA* mutations (Markalunas et al. 2024) ↑ in patients with invasive ductal carcinoma (Markalunas et al. 2024)	Chemotherapy response.(Shafiq et al. 2020)
Galectin-7	↑ increase metastasise ability of BC cells (Demers et al. 2010) ↑ in the cytoplasm—worse outcome (Trebo et al. 2020)	↑ (in stage I) (Blair et al. 2021)	Targeted therapy (cell lines) (Grosset et al. 2014)
Galectin-8	↑ in the cytoplasm—improved outcome (Trebo et al. 2020)	↑ (Barrow, et al. 2011) ↑ in patients with *KIT* mutation (Funkhouser et al. 2022)	Therapeutic potential (Chien et al. 2025)
Galectin-9	Antimetastatic and prognostic potential (Irie, et al. 2005)	↑ in HER2-amplified tissues (Funkhouser et al. 2023) ↓ in samples with positive hormone receptor markers (Funkhouser et al. 2023) ↑ in patients with *KIT* mutation (Funkhouser et al. 2022; Markalunas et al. 2024) ↓ in patients with *FLT3* mutation in luminal A subtype (Markalunas et al. 2024)	Dynamic biomarker after radiotherapy for TNBC, therapeutic implications (Lerévérend et al. 2025)

↑—high expression/concentration, ↓—low expression/concentration

### Adipokines

Adipokines (or adipocytokines) are cytokines (cell signalling proteins) secreted by adipose tissue that consists of adipocytes, immune cells and an extracellular matrix (Kothari et al. 2020; Hoy et al. 2017). Adipocytes constitute the predominant cell population in the breast stroma and participate in the crosstalk with BC cells contributing to proliferation and invasion of BC cells as well as resistance to therapy (Hoy et al. 2017; Christodoulatos et al. 2019; Samuel et al. 2018). Adipocytokines are produced mainly by adipocytes and are associated with obesity, insulin resistance, lipid disorders, endothelial dysfunction, tumour pathogenesis etc., therefore they are involved in the pathogenesis of many diseases affecting different body systems (e.g. diabetes mellitus, gynaecological diseases, rheumatologic disorders or cancers) (Maximus et al. 2020).

Among adipocytokines, the increased levels of leptin and resistin as well as decreased levels of adiponectin are associated with BC development (Li and Han 2018). Another adipokines, such as visfatin, apelin, lipocalin 2, osteopontin or oncostatin M were also linked to BC (Christodoulatos et al. 2019). In this review, we describe the importance of adiponectin, leptin and resistin in BC.

#### Adiponectin

Adiponectin is a glycoprotein that exhibits pleiotropic protective effects, including insulin sensitizing, antiapoptotic, antioxidant, anti-inflammatory and anti-atherogenic properties (Andò et al. 2020; Nehme et al. 2022; Nagaraju et al. 2016). The effects of adiponectin are mediated through its binding to membrane receptors, such as adiponectin receptors 1 (AdipoR1) and 2 (AdipoR2) and T-cadherin in target tissues and organs (Li and Han 2018; Andò et al. 2020).

Adiponectin is considered to have an inhibitory effect on BC development (Nehme et al. 2022). Meta- analysis found the association of decreased serum levels of adiponectin with an increased BC risk in premenopausal and postmenopausal women (Gu et al. Jul. 2018; Yu et al. 2019), while others found an association only between adiponectin and postmenopausal BC (Ye, et al. 2014; Liu et al. 2013). Moreover, BC women with the low serum levels of adiponectin were found to be more likely to present an aggressive phenotype (large tumours, high histological grade) (Miyoshi, et al. 2003). Oh et al. (2011) showed that serum adiponectin levels were higher in ER/PR-positive BC patients. Moreover, in the ER/PR-negative patients serum adiponectin levels showed an inverse association with the risk of BC recurrence. The authors suggest that assessing the concentrations of adiponectin may be useful in establishing prognosis in ER/PR-negative BC and that actions aimed at increasing serum adiponectin levels may protect against recurrence in ER/PR-negative cancers (Oh et al. 2011). Higher expression of adiponectin and lower expression of its receptor ADIPOR1 were found in BC tissue of postmenopausal women with normal BMI in comparison to women with overweight or obesity (Orozco-Arguelles et al. 2021). Jeong et al. (2011) showed that positive expression rates of adiponectin and AdipoR were significantly higher in invasive BC than in patients with ductal carcinoma in situ (DCIS). Moreover, in invasive BC, high expression of adiponectin was associated with lower T-stage, while the expression of AdipoR was associated with high Ki-67 expression. The authors suggest that high expressions of adiponectin and AdipoR may be related to BC invasiveness (Jeong et al. 2011). Several studies demonstrated that in ER-negative BC cells adiponectin inhibits cell growth, induces apoptosis and inhibits cell proliferation (Kang et al. 2005; Grossmann et al. 2008; Santos et al. 2008; Andò et al. 2019). However, it was shown that adiponectin stimulates growth in ER-positive BC cells (Mauro et al. 2014). It was also found that adiponectin may differentially regulate cyclin D1 (CD1) expression in BC cells depend on ER-α expression, what results in the divergent effects of adiponectin on cell growth (Mauro et al. 2015).

#### Leptin

Leptin is a peptide hormone (encoded by the *LEP* gene) produced mainly by adipocytes and secreted in proportion to the mass of adipose tissue. The main known functions of leptin include regulation of appetite and energy homeostasis (Ramos-Lobo and Donato 2017; Buonaiuto et al. 2022; Skoracka et al. 2025). Leptin participate in many physiological functions, such as glucose homeostasis, neuronal development and plasticity, memory or reproduction (Ramos-Lobo and Donato 2017). Binding of leptin with the long isoform of its transmembrane receptor (ObR) activates signalling pathways, such as JAK2/STAT3, MAPK and PI3K/Akt involved in the processes of cell proliferation, differentiation, migration and invasion (Andò et al. 2019).

Wang et al. (2023a) showed high expression of leptin and leptin receptor (LEPR) in BC tissues, while the expression in benign breast tissues was low. Moreover, leptin expression was significantly higher in patients with lymph node metastases than in patients without such metastases, whereas the expression of LEPR was associated with higher Ki-67 rate. Additionally, the authors found that neither leptin nor LEPR had an impact on survival (Wang et al. 2023a). A meta-analysis by Gu et al. (2019) showed significantly higher serum leptin levels in BC patients compared to the healthy controls. Moreover, patients with lymph node metastases had significantly higher serum levels of leptin than cases without those metastases (Gu et al. 2019). The results of another meta-analysis by Niu et al. (2013) revealed that serum leptin levels increased (from low to high) among groups in the following order: healthy people, breast benign cases, BC patients and BC patients with lymph node metastasis. This may suggests leptin’s role in BC development and prognosis (Niu et al. 2013). Pan et al. (2018) showed in their meta-analysis the association between serum leptin levels and BC risk in postmenopausal women and overweight or obese patients (Pan et al. 2018). A study by Koprivčić et al. (2022) demonstrated significantly higher leptin levels in postmenopausal women with either benign or malignant breast tumour compared to premenopausal group. Additionally, the highest leptin levels had postmenopausal obese women in comparison to other postmenopausal women and premenopausal patients (Koprivčić et al. 2022). In premenopausal BC cases, several studies noticed an inverse association between leptin levels and BC risk (Harris et al. 2011; Petridou et al. 2000; Falk et al. 2006), but some studies did not found such an association (Mantzoros et al. 1999; Sauter et al. 2004).

#### Resistin

Resistin is an inflammatory cytokine that belongs to the resistin-like molecule family (RELMs) and is expressed mostly by the macrophages. Binding of resistin to its receptors, such as the transmembrane Toll-like receptor 4 (TLR4) and adenylyl cyclase-associated protein 1 (CAP1) induces activation of diverse signalling pathways leading, for example, to cancer cell proliferation and invasion, inhibition of apoptosis or resistance to therapy (Sudan et al. 2020). Resistin has pro-inflammatory properties and plays a role in the pathogenesis of different malignancies (Sudan et al. 2020). A study on BC cell lines demonstrated that resistin induces epithelial to mesenchymal transition (EMT) and stemness of BC cells leading to the promotion of metastatic potential of BC cells. (Avtanski et al. 2019). In another study resistin was shown to stimulate invasion and migration of BC cells through phosphorylation of ezrin, radixin and moesin (ERM) proteins (Lee et al. 2016). Moreover, resistin was found to induce migration and invasion of BC cells and promote their aggressive phenotype through the activation of STAT3 (Deshmukh et al. 2015).

BC patients exhibit higher concentrations of resistin compared to healthy controls as was shown in a recent meta analysis (Zoroddu et al. 2024). In premenopausal women serum levels of resistin were shown to be inversely related to BC risk as was found by Georgiou et al. (Georgiou et al. 2016). In BC tissue high resistin expression was found to be associated with a more malignant clinicopathological status and worse prognosis (Lee et al. 2012). Similarly, serum resistin was shown to be significantly associated with cancer stage, grade, tumour size, and lymph node invasion in postmenopausal BC (Dalamaga et al. 2013). Coskun et al. (2016) investigated that BC patients with stage II and III had increased levels of resistin after completion of the oncological treatment compared to pre-treatment resistin levels. Moreover, the studies demonstrated that resistin may play a role in promoting chemoresistance in BC cell lines (Liu et al. 2017; Deshmukh et al. 2017).

### Autoantibodies

The production of autoantibodies is triggered when tumour-associated antigens are no longer recognised by immune system as self-antigens (Rauf et al. 2020). As this response to cancer occurs early in tumorigenesis, the detection of tumour autoantibodies should enable earlier cancer diagnosis even before the appearing of clinical symptoms (Rauf et al. 2020; Montero-Calle et al. 2024; Yang et al. 2022).

Among autoantibodies described in BC (reviewed (Rauf et al. 2020; Montero-Calle et al. 2024; Yang et al. 2022)), one of the best diagnostic ability to distinguish between early stage BC patients and benign breast disease or healthy controls possess autoantibodies against TOPO48 (SN 76%, SP 100%, AUC = 0.801) described by He et al. (2020). Moreover, the authors showed improved survival rates for early stage BC patients with positive detection of the anti-TOPO48 autoantibody compared to those with a negative detection of this autoantibody (He et al. 2020). Dong et al. (2013), using phage display, identified another autoantibodies, heterogeneous nuclear ribonucleoproteins F (hnRNPF) (SN 84.2%, SP 60.8%, AUC = 0.725) and ferritin heavy chain (FTH1) (SN 81.2%, SP 56.1%, AUC = 0.686). Many other individual autoantibodies have been studied in BC, such as p53, MUC1, Her2/Neu, c-MYC, NY-ESO-1, IMP2/p62, HSP60, survivin, CDKN2A (p16), BRCA1, BRCA2 or cyclin B1 autoantibody. However, most of the studied autoantibodies possess low sensitivities and their individual assessment is not useful for BC screening (Rauf et al. 2020; Montero-Calle et al. 2024; Yang et al. 2022). Thus, many studies focused on combining autoantibodies into a panel. Selected panels of autoantibodies are presented in Table 2.

**Table 2:** Comparison of the diagnostic utility of autoantibody panels in BC

Autoantibody panels	SN [%]/SP [%]/AUC	References
GAL3+PAK2+PHB2+RACK1+RUVBL1	66/84/0.81	Lacombe et al. (2013)
p-53+c-myc+HER2+NY-ESO-1+BRCA1+BRCA2+MUC1	64/85/-	Chapman et al. (2007)
ANGPTL4+DKK1+GAL1+MUC1+GFRA1+GRN+LRRC15 (+ age, BMI, race, current smoking status)	72.9/76/0.82	Evans et al. (2014)
BMI-1+HSP70+NY-ESO-1+p53	63.4/90.2/0.819	Hong et al. (2021)
RAD50+PARD3+SPP1+SAP30BP+NY-BR-62+NY-CO-58	70/91/0.808	Kostianets et al. (2017)
CyclinB1+Imp1+Koc+survivin+p16+c-myc	67.3/92.2/-	Liu et al. (2015)
CTAG1B+CTAG2+TP53+RNF216+PPHLN1+ PIP4K2C+ZBTB16+TAS2R8+WBP2NL+DOK2+ PSRC1+MN1+TRIM21	33/98/0.68	Wang, et al. (2015)
ATP6AP1+PDCD6IP+DBT+CSNK1E+FRS3+RAC3+ HOXD1+SF3A1+CTBP1+C15orf48+MYOZ2+EIF3E+ BAT4+ATF3+BMX+RAB5A+UBAP1+SOX2+GPR157+ BDNF+ZMYM6+SLC33A1+TRIM32+ALG10+TFCP2+ SERPINH1+SELL+ZNF510	80.8/61.6/0.756	Anderson et al. (2011)

### MicroRNAs (miRNAs)

MicroRNAs (miRNAs) are a major class of non-coding RNAs that regulate gene expression at the mRNA level (Saliminejad et al. 2019; Bartel 2004). It is known that miRNAs play a key role in the carcinogenic process. In BC, miRNA dysregulation may occur through genetic and epigenetic mechanisms (Mulrane et al. 2013).

A recent study by Chekhun et al. (2024) presents miRNA that can constitute potential prognostic and diagnostic BC biomarkers. The study showed that an increase in the expressions of miR-182, miR-21, miR-29b and miR-34a and a decrease in the miR-27a expression in the tumour tissue is associated with high malignancy degree of BC in young women. Moreover, it was shown that the expression of miRNAs was associated with BC molecular subtypes. Higher expression of miR-182 and lower levels of miR-21 and miR-27a were revealed in luminal A cases than those in luminal B tumours. Additionally, the authors showed lower miR-145 and higher miR-29b expression in receptor-negative BC compared to the luminal A subtype and lower expression of miR-27a compared to the luminal B cases. Overexpression of miR-182 and miR-21 was demonstrated in both luminal A and B subtypes (Chekhun et al. 2024).

In another study, blood plasma levels of miR-25, miR-155, miR-27, miR-335, miR-200 and miR-497 were analysed (Harashchenko 2024). The study showed significantly increased levels of miR-27 and miR-335 in postmenopause BC patients. In addition, it was found a trend toward an increase in the miR-155 and miR-200 molecules levels. Moreover, the expression levels of miR-27 and miR-497 were significantly lower in premenopausal patients after adjuvant polychemotherapy, whereas in menopausal patients after neoadjuvant polychemotherapy miR-27 level decreased and miR-497 increased (Harashchenko 2024). Canatan et al. (2021) found that miR-21, miR-155 and miR-125 molecules may be promising circulating biomarker useful in the diagnosis of early-stage BC. The potential utility of serum circulating mi RNAs: miR-21 and miR-155 as BC biomarkers was also demonstrated in another study (Fan et al. 2018). Among other circulating miRNAs studied in BC are, for example, miR-10b, miR-17, miR-30b, miR-103a or miR-200c (Liu et al. 2022).

Elhelbawy et al. (2021) showed that miR-148a and miR-30c expressions were down regulated in BC patients. Moreover, the expression levels of both miRNAs were negatively correlated with CEA and CA15-3 (Elhelbawy et al. 2021). Ahmed et al. (2022) investigated that the diagnostic efficacy for miR-29a and miR-335 were superior to the classic tumour markers CEA and CA15-3 for early detection of BC. In a systematic review by Duque et al. (2022), which analysed biomarkers obtained in liquid biopsy (LB) for early BC diagnosis, it was observed that the analysis of miRNA predominates among LB methods. Table 3 shows selected miRNAs with altered expression in different BC subtypes..

**Table 3:** miRNAs with altered expression in BC subtypes

BC subtype	miRNA Downregulated		miRNA upregulated	References
Luminal A	miR-29a miR-652 miR-181a			McDermott et al. (2014)
	miR-23b miR-181c miR-29a miR-29b miR-181d		miR-142 miR-155 miR-16	Triantafyllou et al. (2022)
	hsa-miR-18b-5p hsa-miR-487b-5p hsa-miR-526b-5p hsa-miR-543 hsa-miR-627-5p hsa-miR-3614-5p hsa-miR-6503-5p		hsa-miR-23a-3p hsa-miR-122-5p hsa-miR-196a-5p hsa-miR-301b-5p hsa-miR-376a-3p hsa-miR-873-3p	Souza et al. (2019)
Luminal B	hsa-miR-128-1-5p		hsa-miR-203a-5p hsa-miR-502-5p hsa-miR-548ar-5p	Souza et al. (2019)
HER2+	miR-101-5p			Normann et al. (2022)
	hsa-miR-584-3p hsa-miR-615-3p		hsa-miR-548ar-5p	Souza et al. (2019)
TNBC			miR-21	Fang et al. (2017b)
			miR-93	Hu et al. (2015)
	miR-145 miR-195		miR-221 miR-21 miR-210 let-7a	Thakur et al. (2016)
			miR-17-92 cluster	Farazi et al. (2011)

**Table 4:** CircRNA, miRNAs and targeted mRNAs in BC

CircRNA	miRNA	mRNA	References
*Promoting cancer development*			
hsa_circ_0007255	miR-375	KIF4A	Tang et al. (2019)
hsa_circ_0000515	miR-296-5p	CXCL10	Cai et al. (2021)
hsa_circ_0072088	miR-578	HIF1A	Chen et al. (2020a)
hsa_circ_0061825	miR-326	TFF1	Pan et al. (2020)
hsa_circ_0007534	miR-593	MUC19	Song and Xiao (2018)
circ_0000511	miR-326	TAZ	Wu et al. (2021)
hsa_circ_002178	miR-328-3p	COL1A1	Liu et al. (2020)
*Inhibiting cancer development*			
circRNA_000554	miR-182	ZFP36	Mao et al. (2020)
hsa_circ_0002018	miR-658	UPK1A	Xu et al. (2020)
hsa_circ_0000320	miR-421	RASA1	Xiao et al. (2019)
hsa_circ_0001785	miR-942	SOCS3	Li et al. (2020a)
circ_0001368	miR-1204	ALX4	Yi et al. (2022)
hsa_circ_0001098	miR-3942-3p	BARD1	Zhao et al. (2018)
hsa_circ_0001451	miR-197-3p	FBXW7	Ye et al. (2019)

Circular RNAs (circRNAs) are noncoding, single-stranded RNAs formed in a circular structure (Chen and Shan 2021). CircRNA has the ability to serve as miRNA sponge by binding circRNA with miRNA and thus inhibit miRNA activity. This results in the regulation of target genes expression (Chen and Shan 2021). The circRNA-miRNA-mRNA regulatory network can participate in the processes associated with promotion and inhibition of BC, such as proliferation, migration, invasion and chemotherapy resistance (Zhang et al. 2021b). Thus, circRNA-miRNA-mRNA axis is considered to be a promising biomarker in the diagnosis and prognosis of BC (Zhang et al. 2021b). Table 4 presents selected circRNAs, miRNAs and target mRNAs that play a role in BC promotion and inhibition.

Despite the great interest and progress in the detection of circRNA, its analysis remains a challenge due to its characteristics, such as circular structure or low abundance. Northern blotting, that is considered the gold standard method for validating circRNA, is limited because of its low sensitivity, involvement of multiple and time-consuming steps or low throughput (Mi et al. 2022; Feng et al. 2023). The establishment of standard detection methods is a need to use circRNAs for diagnosis and treatment (Ali et al. 2025).

### ctDNA

Circulating tumour DNA (ctDNA) occurs mostly as160–200 base pairs in length fragments and constitutes a distinctive subset of cell-free DNA (cfDNA) (Wang et al. 2023b). It is thought that ctDNA originates from tumour cells through apoptosis, necrosis and active secretion from cellular structures, but it could also arise from circulating tumour cells (CTCs) (Dao et al. 2023; Sant et al. 2022). Thus, ctDNA can reflect BC heterogeneity and its analysis may be useful in early diagnosis, the detection of minimal residual disease (MRD) and early relapse as well as monitor tumour evolution and selection of treatment (Sant et al. 2022).

Promising results were obtained by Zhang et al. (2019), who showed in early-stage BC patients who need chemotherapy that ctDNA may serve as a sensitive and specific biomarker for BC diagnosis. Additionally, positive ctDNA in patients after surgery may suggest possible recurrence and metastasis (Zhang et al. 2019). Similarly, Rodriguez et al. (Rodriguez et al. 2019), comparing PIK3CA and TP53 mutations between breast tumour tissues and ctDNA in blood samples collected before biopsy, demonstrated that analysis of ctDNA may be useful in early BC diagnosis. Moreover, the authors showed the association between the presence of ctDNA mutations and more aggressive clinicopathological features (Rodriguez et al. 2019). The usefulness of ctDNA in detection of recurrence in BC was also performed. Coombes et al. (Coombes et al. 2019) revealed the ability of ctDNA assay to predict BC recurrence earlier than CT imaging, CA 15–3 measurements and liver function tests (Coombes et al. 2019). Moreover, the association of elevated plasma ctDNA with a poor outcomes in early and metastatic BC patients and high risk of relapse was shown in a meta-analysis (Cullinane et al. 2020). Papakonstantinou et al. (2022) in a systematic review and meta-analysis found that the presence of ctDNA at baseline and after completion of neoadjuvant therapy was associated with a worse survival and a higher risk of relapse. Moreover, ctDNA analysis is a promising and minimally invasive method, in comparison with tissue-based testing, for identifying estrogen receptor alpha gene (ESR1) mutations, which drive endocrine therapy resistance in patients with HR+/HER2- metastatic BC (Venetis et al. 2023). Li et al. (2020b) showed that using ctDNA to dynamic monitoring of ESR1 mutations could be a biomarker in prediction of endocrine resistance in ER+ MBC patients.

Some studies analysed ctDNA in other specimens, such as urine, cerebrospinal fluid (CSF) or breast milk (BM). Guan et al. (2020) observed higher urinary DNA in early BC patients compared to healthy individuals. Moreover, urinary DNA showed clinical relevance in BC (Guan et al. 2020). Liu and Liu (2018) demonstrated that urinary DNA may be useful in predicting relapse in BC patients. Siravegna et al. (2017) found that in HER2-positive MBC patients with brain metastases paired plasma and CSF ctDNA analysis may be useful in the management of these women. Saura et al. (2023) demonstrated the presence of ctDNA in BM collected from patients with early BC occurring during pregnancy and postpartum. Additionally, the authors showed that in early BC a superior source of ctDNA is BM compared to plasma (Saura et al. 2023).

The analysis of ctDNA in clinical use has several limitations. Degradation of DNA fragments during sampling and storage may result in its low levels for ctDNA analysis in the circulation. Thus, the detection of ctDNA requires high sensitive methods. Moreover, there is a lack of established standardization for sample collection and analysis of ctDNA (Parums 2025). The analytical methods used for ctDNA analysis include, for example, polymerase chain reaction (PCR) or next-generation sequencing (NGS) methods (Parums 2025). The emerge ultrasensitive method for ctDNA analysis in BC patients is whole genome sequencing (WGS) (Parums 2025; Garcia-Murillas et al. 2025; Vavoulis et al. 2025).

### Circulating tumour cells (CTCs)

CTCs are cancer cells disseminated from the primary tumour that enter the bloodstream and appear as single CTC or CTC clusters possessing enhanced metastatic potential (Cani and Hayes 2024; Chauhan et al. 2021; Zhang et al. 2023; Zhang et al. 2021c; Lin et al. 2021). Their isolation is challenging, as CTCs are heterogeneous and very rare in the blood (Cani and Hayes 2024; Chauhan et al. 2021; Zhang et al. 2023; Lin et al. 2021). As the proportion of these cells is extremely low in comparison with other cells present in the blood, the enrichment of CTCs is required for their isolation (Chauhan et al. 2021). Various methods developed for the isolation of CTCs are based on their physical (e.g. size, density) and biological (marker depended) properties (Chauhan et al. 2021; Zhang et al. 2023; Lin et al. 2021). The techniques that are used predominantly are based on the isolation of CTCs expressing epithelial cell adhesion molecule (EpCAM) (Lin et al. 2021; Perelmuter et al. 2024). However, the use of combination techniques may improve CTCs isolation (Rzhevskiy et al. 2025).

In early BC, CTCs are detected in approximately 20–30% of patients and about 60% of advanced BC cases (Perelmuter et al. 2024; Fabisiewicz et al. 2020). Numerous studies investigated the potential prognostic significance of CTCs in both early as well as metastatic BC. In early BC, the presence of CTCs in the blood is rare, thus their use as a biomarker is limited in that population (Fabisiewicz et al. 2020; Thomas-Bonafos et al. 2024). Nevertheless, a meta-analysis by Bai et al. (2023) showed that CTCs have diagnostic value in BC. Moreover, the prognostic potential of CTCs were investigated in neoadjuvant and adjuvant studies, which found association between the presence of CTCs and worse prognosis in early BC (Cani and Hayes 2024; Matikas et al. 2022; Bidard et al. 2018).

In MBC, it was found that using a CTC threshold of 5 cells per 7.5 ml allows stratification of patients into two subgroups: Stage IV indolent group (< 5 CTCs/7.5 ml) having a favourable prognosis and Stage IV aggressive group (≥ 5 CTCs/7.5 ml) having a poor prognosis (Thomas-Bonafos et al. 2024; Cristofanilli et al. 2019). In longitudinal analysis of CTCs in MBC patients by Szostakowska-Rodzos et al. (2024) confirmed the prognostic value of high CTC count (≥ 5 CTCs) and presented that the dynamics of CTCs during treatment may predict cancer progression. The clinical utility of CTCs in MBC has been investigated in phase II and phase III randomized trials (Thomas-Bonafos et al. 2024).

### Extracellular vesicles (EVs)

EVs are nano-sized membrane-enclosed vesicles that contain various biomolecules, including proteins, lipids and nucleic acids and play a significant role in intracellular communications (Lee et al. 2023a). EVs are differentiated as: exosomes (small EVs; 50–150 nm in diameter), microvesicles (large EVs; 100–1000 nm) and apoptotic bodies (500–5000 nm) (Lee et al. 2023b). Exosomes are secreted by all cell types and carry biomolecules (proteins, lipids, messenger RNAs, microRNAs, long noncoding RNAs, DNAs) from their cell of origin, having potential to be a great source of tumour biomarkers (Maia et al. 2018; Urabe et al. 2020). The presence of EVs was demonstrated in biofluids, such as blood, urine, saliva, breast milk, cerebrospinal fluid and ascites (Lee et al. 2023b).

EVs were found to have the potential to differentiate molecular subtypes of BC by analysing subtype-specific EV protein signatures or molecular features of cancer exosomes (Lee et al. 2023a; Rontogianni et al. 2019; Cao et al. 2022). Xu et al. (2024) investigated that the protein analysis of serum EVs in BC patients may serve as a minimally invasive tool for early detection of BC and the assessment of lymph node metastasis. Moreover, it was found that TALDO1 may serve as an EV serum biomarker for MBC (Xu et al. 2024). Additionally, Dorado et al. (Dorado et al. 2024) showed that the lipid composition of blood plasma EVs have the potential for BC detection. EVs were also found to have the roles in mediating BC therapy resistance (Samuels et al. 2022).

## Combination of biomarkers in BC

Markers with higher sensitivity and specificity, useful in BC diagnosis and management are still being sought. Several studies combined commonly used biomarkers with new, potential biomarkers to achieve better clinical utility.

Several studies investigated the usefulness of different combinations of classical tumour markers, such as CA15-3 and CEA, with selected parameters in early diagnosis of BC. Chen et al. (2020b) compared the sensitivities and specificities of single tests: CEA, CA 15–3, CA125 (SN 4.89%, SP 95.02%) and tumour abnormal protein (TAP) (SN 4.89%, SP 99.62%), and in combinations for the detection of BC. The Authors found that the combination of those four tests, TAP+CEA+CA125+CA15-3, achieved the highest sensitivity (21.84%) for the diagnosis of BC. However, the high specificities had TAP+CEA+CA15-3 (SP 97.70%) combination and it might be helpful for confirming BC. Among the combinations without TAP, the highest sensitivity had CEA+CA125+CA15-3 combination (SN 16.95%) and the highest specificity had CEA+CA15-3 (SP 98.08%) (Chen et al. 2020b). Luo et al. (2022) assessed the diagnostic value of different combinations of common tumour markers: alpha fetoprotein (AFP), carbohydrate antigen 19–9 (CA19-9), CEA, CA15-3 and CA125. The Authors found that among combinations of three parallel detections of BC tumour markers, AFP+CEA+CA15-3 had the highest sensitivity (SN 83.46%) and CEA+CA125+CA19-9 had the highest AUC (AUC = 0.922). Moreover, AFP+CEA+CA15-3 and AFP+CA15-3+CA125 had the highest accuracy (80.25%) (Luo et al. 2022). Wang et al. (Wang et al. 2014) analysed a panel of four potential serum/nipple discharge biomarkers: CEA, CA15-3, CA125 and tumour-specific growth factor (TSGF) in the diagnosis and prognosis of BC. The study showed that the combination of those four serum/nipple-discharge markers had significantly higher AUC value and diagnostic sensitivity in comparison to the four markers in serum, in nipple discharge or individual markers (Wang et al. 2014).

Zou et al. (2022) analysed routine tests indicators: neutrophil-to-lymphocyte ratio (NLR), red cell distribution width (RDW) and serum tumour markers (CEA, CA15-3, CA125, CA19-9) in BC and breast fibroadenoma (FA). NLR, CEA and CA19-9 values showed statistically significant differences between BC and FA patients and the study revealed significantly higher diagnostic efficiency of combined indicators than of the single parameters: CEA (SN 81.8%, SP 69.4%, AUC = 0.799), NLR (SN 74.2%, SP 69.4%, AUC = 0.747), CA19-9 (SN 70.0%, SP 61.1%, AUC = 0.711). The combination of three parameters: NLR+CEA+CA19-9 (SN 76.5%, SP 88.9%, AUC = 0.886; 95% confidence interval: 0.838–0.933, *P* = 0.000) showed the best diagnostic efficacy and the authors concluded that this combination may be used to screen and diagnose BC (Zou et al. 2022). Zuo et al. (2016) screened complex autoantigens in the serum of BC patients using serological analysis of recombinant cDNA expression libraries (SEREX) and phage display technology. The authors identified a panel of autoantigens, including lectin, galactoside-binding, soluble 3 (LGALS3; SN 63%, AUC = 0.687), prohibitin 2 (PHB2; SN 43%, AUC = 0.583), mucin 1 (MUC1; SN 46%, AUC = 0.563) and glycerol kinase 2 (GK2; SN 46%, AUC = 0.608) that in combination with CA15-3 improved sensitivity, specificity and overall survival in the diagnosis of BC (stage T_1_N_0_M_0_). SP of each individual antigen were not less than 60% and AUCs were less than 0.8 (Zuo et al. 2016). Dong et al. (2013) identified two novel autoantibody biomarkers, hnRNPF and FTH1. The authors showed that the analysis of these two autoantibody biomarkers: hnRNPF (SN 84.2%, SP 60.8%, AUC = 0.725) and FTH1 (SN 81.2%, SP 56.1%, AUC = 0.686) in combination with CA15-3 improved sensitivity and specificity (Dong et al. 2013). Liu et al. (2018) demonstrated that the panel containing programmed cell death protein 1 (PD-1), interleukin-10 (IL-10), interleukin-2 receptor alpha (IL-2Rα) and CA15-3 can discriminate BC from benign breast disease. Moreover, this panel showed the highest AUC (0.811) for early-stage BC discrimination, while a panel of PD-1, IL-10 and CA15-3 had the highest AUC (0.896) for advanced BC discrimination (Liu et al. 2018). Zajkowska et al. (2019) analysed the members of vascular endothelial growth factor receptor (VEGF) family: VEGF-A, VEGF-C, VEGF-D and their receptor VEGFR-2 individually and in combination with CA 15–3. The study showed that among all tested parameters in the total BC group VEGF-C had the highest AUC (0.7672) as well as in stage I (AUC = 0.7684) and stage II (AUC = 0.7772) of BC. Moreover, the combination of CA 15–3 and VEGF-C was the most favourable in total BC group (Zajkowska et al. 2019). The diagnostic utility of single CEA and CA15-3 assays and their selected combinations with other parameters in diagnosis of BC are shown in Table 5.

**Table 5:** Comparison of the diagnostic utility of single CA15-3 and CEA assays and selected combinations of these markers with other parameters in the diagnosis of BC patients

Parameter	SN [%]	SP [%]	AUC	References
CEA	7.18	98.85	–	Chen et al. (2020b)
CA15-3	7.47	99.23	–	
CA125	4.89	95.02	–	
CEA+CA15-3	12.93	98.08	–	
CEA+CA125+CA15-3	16.95	93.10	–	
TAP+CEA+CA15-3	17.82	97.70	–	
TAP+CEA+CA125+CA15-3	21.84	92.72	–	
CEA	23.94	67.44	0.679	Luo et al. (2022)
CA15-3	15.00	96.43	0.727	
CA125	16.54	93.57	0.695	
AFP+CEA+CA15-3	83.46	74.29	0.913	
CEA+CA125+CA19-9	83.08	73.57	0.922	
AFP+CA15-3+CA125 (accuracy 80.25%)	82.31	77.14	0.905	
*Nipple discharge (ND)*				Wang et al. (2014)
CEA	69.8	86.0	0.779	
CA15-3	74.4	82.4	0.784	
CA125	72.1	83.8	0.780	
*Serum*				
CEA	53.5	89.0	0.712	
CA15-3	60.5	91.9	0.762	
CA125	55.8	90.4	0.732	
(ND+serum) CEA+CA15-3+CA125+TSGF	97.7	75.0	0.863	
CEA	81.8	69.4	0.799	Zou et al. (2022)
NLR+CEA+CA19-9	76.5	88.9	0.886	
CA15-3	58	-	0.634	Zuo et al. (2016)
CA15-3+LGALS3+PHB2+MUC1+GK2	87	76	0.872	
CA15-3	69.1	89.4	0.792	Dong et al. (2013)
FTH1+CA15-3	85.1	92.7	0.834	
hnRNPF+CA15-3	87.4	91.0	0.862	
FTH1+hnRNPF+CA15-3	89.3	93.8	0.931	
*early-stage BC*				Liu et al. (2018)
CA15-3	52.3	73.3	0.607	
PD-1+IL-10+IL-2Rα+CA15-3	93.3	61.4	0.811	
*advanced BC*				
CA15-3	65.2	100	0.791	
PD-1+IL-10+CA15-3	93.3	78.3	0.896	
*BC (total)*				
CA15-3	64.4	73.3	0.707	
PD-1+IL-10+IL-2Rα+CA15-3	93.3	72.4	0.862	
CA15-3	58.33	95.00	0.7573	Zajkowska et al. (2019)
VEGF-C+CA15-3	86.67	70.00	0.8476	

Different combinations of biomarkers have also been studied in metastatic breast cancer (MBC). Diagnostic utility of selected combinations of CEA and CA15-3 with other biomarkers in MBC is shown in Table 6.

**Table 6:** Comparison of the diagnostic utility of single CA15-3 and CEA assays and combinations of these markers in the diagnosis of metastatic BC (MBC)

Biomarker	SN [%]	SP [%]	AUC	References
*(MBC vs. BC without mestastases)*				Wang et al. (2017)
CEA	56.7	92.0	0.806	
CA15-3	44.5	84.5	0.743	
CEA+CA 15–3	68.9	88.0	0.817	
CEA+TPS	78.7	82.0	0.863	
CA15-3+CA125	52.4	91.5	0.708	
*(Patients with distant metastasis)*				Zhang et al. (2021d)
CEA	57.1	87.4	0.755	
CA15-3	59.2	94.1	0.821	
CEA+CA15-3	81.6	83.8	0.855	
CEA+CA 15–3+TPS	83.7	79.4	0.866	
TPS+CA15-3+CEA+CA125	85.7	78.2	0.846	
*(patients with occult metastasis in three years)*				
CEA	39.7	94.8	0.725	
CA15-3	47.9	94.0	0.744	
CEA+CA15-3	60.3	83.9	0.764	
CA 15–3+TPS	57.5	91.0	0.799	
CEA+CA 15–3+TAP	57.5	91.0	0.799	
CA 15–3+CA 125+TAP	57.5	91.0	0.799	
TPS+CA15-3+CEA+CA125	57.5	91.0	0.799	

TPS, tissue polypeptide specific antigen

Classic tumour markers were also studied in combinations with miRNA. Zaleski et al. (2018) in the comparison of BC and healthy controls found that the combination of miR-34a (SN 36.2% and 34.0% at 90% and 95% SP, AUC = 0.722) with CEA or CA15-3 improved the performance with AUCs of 0.844 and 0.800 respectively. The combination of CEA and CA15-3 reached only slightly higher performance (AUC = 0.741, SN of 38.5% at 90% and 95% SP). When BC was compared with benign breast diseases, the combination of miR-34a (SN 36.2% and 34.0% at 90% and 95% SP, AUC = 0.719) with CEA or CA15-3 also resulted in improved performance (AUCs 0.794 and 0.741 respectively) (Zaleski et al. 2018). Similarly, Raheem et al. (Raheem et al. 2019) found that in the differentiation between BC and healthy control group, combination of miRNA-34a and CA15-3 showed a very good discriminative value for BC patients. Qiao et al. (2024) investigated that serum exosomal miR-200c (SN 86.27%, SP 74.47%, AUC = 0.854) can distinguish patients with and without BC. Moreover, combination of miR-200c with traditional serum markers (CEA, CA15-3, CA125) may improve diagnostic efficacy for BC (Qiao et al. 2024) (Table 7).

**Table 7:** Comparison of the diagnostic utility of single CA15-3 and CEA assays and selected combinations of these markers with miRNAs in diagnosis of BC patients

Parameter	SN [%]	SP [%]	AUC	References
(*healthy controls vs BC)*				Zaleski et al. (2018)
CEA	26.0/18.0	90.0/95.0	0.717	
CA15-3	36.2/31.9	90.0/95.0	0.721	
miR-34a+CEA	59.1/34.1	90.0/95.0	0.844	
miR-34a+CA15-3	56.1/56.1	90.0/95.0	0.800	
*(benign breast diseases vs BC)*				
CEA	20.0/20.0	90.0/95.0	0.623	
CA15-3	25.5/23.4	90.0/95.0	0.619	
miR34a+CEA	54.5/31.8	90.0/95.0	0.794	
miR34a+CA15-3	53.7/53.7	90.0/95.0	0.741	
CA15-3	80	73.3	0.829	Raheem et al. (2019)
miRNA-34a+CA15-3	83.3	77.7	0.842	
CEA	32	100	0.615	Qiao et al. (2024)
CA15-3	53.19	91.49	0.727	
miR-200c+CEA+CA15-3+CA125	91.49	76.6	0.9140	

In addition, many combinations of miRNAs were investigated in the diagnosis of BC. Selected studies were summarised in Table 8.

**Table 8:** Combinations of miRNAs in the diagnosis of BC

Combinations of miRNA	SN [%]	SP [%]	AUC	References
miR-1246+miR-206+miR-24+miR-373	98.0	96.0	0.992	Jang et al. (2021)
miR-145-5p+miR-191-5p (miR-222-3p normalization)	94.3	100	0.984	Ashirbekov et al. (2020)
let-7b-5p+miR-122-5p+miR-146b-5p+miR-210-3p+miR-215-5p (external validation set)	94.4	88.9	0.978	Li et al. (2019)
miR-92a-3p+miR-23b-3p+miR-191-5p	89.2	96	0.977	Sharifi et al. (2022)
miR-1246+miR-1307-3p+miR-4634+miR-6861-5p+miR-6875-5p (in the test cohort)	97.3	82.9	0.971	Shimomura et al. (2016)
miR-145+miR-425-5p+miR-139-5p+miR-130a	97	91	0.97	Itani et al. (2021)
miR-1246+miR-202+miR-21+miR-219B (patients under the age of 50)	85.29	93.33	0.961	Jang et al. (2021)
miR-185-5p+miR-362-5p	92.65	92.31	0.957	Zhang et al. (2021)
miR-451+miR-148a+miR-27a+miR-30b	94.7	82.8	0.953	Luo et al. (2014)
miR-10b-5p+miR-133a-3p+miR-155-3p+miR-195-5p+miR-195-3p	79	100	0.948	Jing et al. (2024)
c-miR-16+c-miR-21+c-miR-155+c-miR-195	88.89	86.67	0.936	Fan et al. (2018)
miR-21+miR-155+miR-365	85.71	85.72	0.918	Han et al. (2017)
miR-199a+miR-29c+miR-424	77.2	88.9	0.905	Zhang et al. (2015)
BC vs normal hsa-miR-324-3p/hsa-miR-382-5p, hsa-miR-21-3p/hsa-miR-324-3p, hsa-miR-30a-5p/hsa-miR-30e-5p, hsa-miR-221-3p/hsa-miR-324-3p	89.0	92.5	0.901	Fang et al. 2018)
BC vs benign hsa-miR-30a-5p/hsa-miR-382-5p, hsa-miR-192-5p/hsa-miR-382-5p, hsa-miR-192-5p/hsa-miR-574-5p, hsa-miR-21-3p/hsa-miR-221-3p, hsa-miR-221-3p/hsa-miR-30a-5p	88.1	77.5	0.901	
BC vs control (normal+benign) hsa-miR-30e-5p/hsa-miR-382-5p, hsa-miR-221-3p/hsa-miR-324-3p, hsa-miR-30a-5p/hsa-miR-382-5p, hsa-miR-152/hsa-miR-382-5p, hsa-miR-192-5p/hsa-miR-382-5p	71.7	78.2	0.820	
miR-195+miR-210+miR-21+miR-16	71.4	100	0.898	Miranda et al. (2024)
miR-21-3p+miR-21-5p+miR-99a-5p	97.9	73.5	0.895	Yu et al. (2018)
EV-miRNAs: miR-9+miR-16+miR-21+miR-429	96.8	80	0.88	Kim et al. (2021)
EV-miRNAs: miR-142-5p+miR-320a+miR-4433b-5p (BC patients vs healthy controls)	93.33	68.75	0.8387	Ozawa et al. (2020)
(luminal A vs healthy controls)	100	93.8	0.9410	
miR-30b-5p+miR-99a-5p	57.4	87.54	0.77	Adam-Artigues et al. (2021)
in stage I BC	82.35	87.54	0.9273	
miR-210+miR-152	83.33	68.0	0.754	Lopes et al. (2021)
miR-23a-3p+miR-130a-5p+miR-144-3p+miR-148a-3p+miR-152-3p	86.5	45.9	0.699	Li et al. (2020)
miR-15a+miR-18a+miR-107+miR-133a+miR-139-5p+miR-143+miR-145+miR-365+miR-425	83.3	41.2	0.665	Kodahl et al. (2014)

## Conclusions

High incidence and mortality rates of breast cancer are prompting researchers to search for new markers that could be of significant diagnostic and prognostic value in breast cancer. Combining traditional diagnostic methods with new markers could improve the effectiveness of diagnosis and monitoring of breast cancer patients and contribute to the implementation of personalised treatment. Ongoing research points to the potential importance of both immunological and molecular markers in breast cancer, as well as the creation of panels of new and traditional markers. Perhaps this approach will prove to be the future of modern breast cancer diagnosis and treatment. However, standardisation for samples collection and methods of novel circulating biomarkers detection is required. Multicentre cohort studies should be prioritised to validate novel biomarkers and their application in clinical practice. Moreover, there is a great need for longitudinal studies to assess the dynamics of biomarkers during therapy.

## Data Availability

No datasets were generated or analysed during the current study.

## Abbreviations

BC: Breast cancer
ER: Oestrogen receptor
PR: Progesterone receptor
HER2: Human epidermal growth factor receptor 2
AI: Aromatase inhibitors
SERMs: Selective ER modulators
SERDs: Selective ER downregulators
IHC: Immunohistochemistry
ISH: In situ hybridization
ADC: Antibody-drug conjugates
CK: Cytokeratin
TPA: Tissue polypeptide antigen
TPS: Tissue polypeptide-specific antigen
AdipoR1: Adiponectin receptor 1
AdipoR2: Adiponectin receptor 2
CA 15-3: Cancer antigen 15–3
CEA: Carcinoembryonic antigen
CA 27-29: Cancer antigen 27–29
CA 125: Cancer antigen 125
DCIS: Ductal carcinoma in situ
LEPR: Leptin receptor
SN: Sensitivity
SP: Specificity
AUC: The area under the curve
miRNA: MicroRNA
circ-RNA: Circular RNA
ctDNA: Circulating tumour DNA
cfDNA: Cell-free DNA
CTCs: Circulating tumour cells
MRD: Minimal residual disease
CSF: Cerebrospinal fluid
BM: Breast milk
TAP: Tumour abnormal protein
AFP: Alpha fetoprotein
CA 19-9: Carbohydrate antigen 19–9
TSGF: Tumour-specific growth factor
NLR: Neutrophil-to-lymphocyte ratio
RDW: Red cell distribution width
MBC: Metastatic breast cancer
